# The pluripotency factor LIN28B is involved in oral carcinogenesis and associates with tumor aggressiveness and unfavorable prognosis

**DOI:** 10.1186/s12935-015-0252-7

**Published:** 2015-10-15

**Authors:** Dongmiao Wang, Yuming Zhu, Yanling Wang, Zhongwu Li, Chunping Yuan, Wei Zhang, Hua Yuan, Jinhai Ye, Jianrong Yang, Hongbing Jiang, Jie Cheng

**Affiliations:** Department of Oral and Maxillofacial Surgery, Nanjing Medical University, Nanjing, 210029 China; Jiangsu Key Laboratory of Oral Disease, Nanjing Medical University, Nanjing, 210029 China; Department of Oral and Maxillofacial Pathology, Nanjing Medical University, Nanjing, 210029 China

**Keywords:** Oral cancer, LIN28B, Oral carcinogenesis, Tumor biomarker

## Abstract

**Objective:**

LIN28B is a conserved RNA-binding protein critically involved in development, cellular metabolism and tumorigenesis. It is frequently overexpressed in human cancers and correlates with tumor aggressiveness as well as unfavorable prognosis. However, the expression pattern and oncogenic roles of LIN28B during oral squamous cell carcinoma (OSCC) development and progression has not been well established yet. Here, we sought to determine the expression of LIN28B and its clinical significance using chemical-induced OSCC animal model, cell lines and primary specimens.

**Method:**

The OSCC animal model was induced using 7,12-dimethyl-1,2-bezan-tracene (DMBA) painting in the hamster buccal pouch. Buccal lesions from animals were obtained from different time points and subjected to routine histological analyses and immunohistochemical staining of LIN28B. The mRNA, protein abundance and subcellular localization of LIN28B was determined in a panel of OSCC cell lines by real-time RT-PCR, western blot and immunofluorescence. The expression levels of LIN28B in human primary OSCC samples were further evaluated by immunohistochemical staining. Moreover, the relationship between LIN28B and several clinicopathological parameters as well as patients’ prognosis were also assessed.

**Results:**

Our results revealed that negative or low LIN28B expression was commonly observed in normal epithelial, whereas more LIN28B abundance was identified in epithelial dysplasia and invasive SCC in the DMBA-induced OSCC animal model. Overexpression of LIN28B was identified in a major fraction of OSCC samples(39/58) and significantly associated with tumor size (*P* = 0.049) and advanced clinical stages (*P* = 0.0286). Patients with increased LIN28B had markedly reduced overall survival as compared to those with low LIN28B. Multivariate survival analyses further indicated that LIN28B abundance served as an independent prognostic factor for patients’ overall survival.

**Conclusions:**

Our findings reveal that LIN28B is critically involved in OSCC initiation and progression and aberrantly overexpressed in human OSCC. It might represent a novel diagnostic and prognostic biomarker for oral cancer.

## Background

Oral squamous cell carcinoma (OSCC) is one of the common solid cancers worldwide with well-known etiologic factors including smoking abuse, alcohol consumption as well as HPV infection, etc. [1]. Despite significant progress in the combined and sequential treatment for OSCC in the last decades, however, the long-term survival rate has not been remarkably improved [2]. Much efforts have been made to unravel the genetic and epigenetic abnormalities behind OSCC pathogenesis and enable us to capture the mechanistic insights into oral cancer progression [3]. However, until now, few biomarkers have been unequivocally established for diagnostic and prognostic management of oral cancer. This unmet challenge significantly impedes the improvement of patients’ treatment outcomes [4]. Therefore, the novel biomarkers discovery and validation is urgent and of great importance for early detection, novel therapeutics development, ultimately leading to improved prognosis.

LIN28B and its mammalian homologue LIN28 are two RNA-binding protein emerged as key modulators for stem cell maintenance, cellular metabolism as well as tumorigenesis [5, 6]. LIN28B and LIN28, initially identified as key regulators of developmental timing in *Caenorhabditis elegans*, were known as master cell reprogramming factors to induce pluripotency in conjunction with KLF4, OCT4 and Sox2 [7, 8]. Mounting evidence has revealed that LIN28B/LIN28 functions as negative regulators of microRNA biogenesis, selectively represses *let*-*7* family production in diverse physiological and pathological settings, although they act in different ways [9]. Moreover, they have been implicated in tumorigenesis and usually aberrantly overexpressed in multiple human cancers including hepatocellular, colon, intestinal and head neck cancers. Their overexpression commonly associated with tumor aggressive behaviors, advanced clinicopathological features, and decreased survival [10–13]. Gain-and loss-of-function studies have further revealed that LIN28B/LIN28 promotes cancer cell proliferation, invasion and metastasis both in vitro and in vivo largely through *let*-*7* repression [10, 12, 14]. Noticeably, comprehensive analyses of LIN28 and LIN28B expression in human cancers pointed to LIN28B as the more relevant homologue underlying tumorigenesis [15]. Further evidence linking LIN28B to tumorigenesis derived from several previous findings that it facilitated cellular malignant transformation in vitro [14–16] and transgenic overexpression was sufficient to initiate neuroblastoma and hepatoblastoma and also required for their maintenance in murine models [11, 17]. Moreover, conditional LIN28B deletion or siRNA-mediated LIN28B knockdown reduced tumor burden, inhibited metastasis and prolonged survival in vivo [10, 18]. Collectively, these findings have provided strong evidence that LIN28B is a bona fide oncogene mediating cancer initiation and progression, and also a novel therapeutic target against cancer.

Accumulating evidence has revealed essential clues regarding LIN28B expression and potential roles in oral cancers [19, 20]. We have revealed a functional single nucleotide polymorphism [variant allele (T) of rs221636] in LIN28B,which is significantly associated with oral cancer susceptibility in a Chinese population, thus supporting the key roles of LIN28B during oral tumorigenesis [21]. However, the expression pattern of LIN28B and its clinicopathological significance in OSCC have not been definitively established yet. In the present study, we sought to determine the LIN28B expression in OSCC cell line, animal model and primary human OSCC specimens, and further identify potential relationships between its abundance and clinicopathological features as well as patients’ prognosis.

## Methods

### Cell lines and chemicals

A panel of human OSCC cell lines including HN4, HN6, Tca8113, Cal27, SCC9, and SCC25 were utilized here. HN4, HN6, Tca8113 and human immortalized oral epithelial cells (HIOEC) were gifts from Prof. Wantao Chen (Shanghai Jiaotong University) [22]. The Cal27, SCC9 and SCC25 were commercially purchased from American Type Culture Collection (ATCC). HIOEC was grown in EpiLife medium (Life technologies) with the addition of human keratinocyte growth supplement (Life technologies). All cancerous cell lines were grown in DMEM/F12 (Life technologies) supplemented with 10 % FBS (Hyclone) and penicillin and streptomycin (100 units/ml), and maintained in a humidified incubator with 5 % CO_2_ at 37 °C.

The carcinogenic chemical polycyclic aromatic hydrocarbon 7,12-dimethyl-1,2-bezan-tracene (DMBA) was purchased from Sigma Aldrich (D3254) and dissolved in mineral oil. Other chemical agents were all purchased from Sigma Aldrich unless otherwise stated.

### Chemical-induced buccal OSCC animal model

The DMBA-induced hamster buccal pouch squamous cell carcinogenesis model was performed with minor modifications as described before [23, 24]. This animal experimental protocol was institutionally approved and conducted in accordance with the Institutional Guide for the Care and Use of Animals. Briefly, forty Syrian golden hamsters (male, 6–8 weeks old, approximately 100–120 g weight) were purchased from Shanghai Laboratory Animal Center, and then randomly divided into three experimental and one control groups (10 animals per group). After environment acclimatization for 1 week, left pouches of animals in the experimental groups were painted with 0.5 % DMBA solution using a No. 4 sable-hair brush on every Monday, Wednesday and Friday for consecutive 16 weeks. The control hamsters were received vehicle only in the similar way. At these following time points (4, 10, 16 weeks), the animals were sacrificed and left buccal pouches were harvested and processed as scheduled.

### Cell immunofluorescence assay

For cellular immunoflurescent staining assay, single cells were pre-seeded and grown on glass coverslips overnight, then fixed with 4 % paraformaldehyde and permeabilized in 0.1 % Triton X-100 and sequentially treated with 3 % BSA and primary antibody against LIN28B (Abcam, ab71415, 1:100 dilution). Following overnight incubation, these cells were further incubated with appropriate secondary antibody and cytoskeleton actin/nuclear DAPI staining. Immunofluorescence was visualized under a Zeiss fluorescence microscope and image-captured using the same parameters.

### RNA extraction and real time RT-PCR

Total RNA was extracted from cells with Trizol (Life technologies) reagent and then subjected to RT-PCR reactions using PrimeScript™ RT-PCR kit (Takara) on a LightCycler 96 device (Roche) as described previously [25]. The gene-specific primers for human LIN28B and GAPDH were used as follows: LIN28B Forward: GCACATTAGACCATGCGAGC; Reverse: CTTTGCTAGCCCCGCCTTC; GAPDH Forward: AGGTGAAGGTCGGAGTCAAC; Reverse: AGTTGAGGTCAATGAAGGGG. Relative mRNA expression was quantified using comparative CT method as compared to GAPDH.

### Protein extraction and western blot

Cells were lysed with ice-cold RIPA buffer supplemented with the protease inhibitor cocktail (Roche). Lysates were resolved by SDS-PAGE, and transferred onto PVDF membranes (Bio-Rad). After incubation with primary antibodies (LIN28B, Abcam, ab71415, 1:1000 dilution; GAPDH, Abcam, ab37168, 1:2000 dilution) overnight, these blots were detected with appropriate secondary antibodies (Life technologies) and visualized by enhanced chemiluminescence (Pierce). The relative levels of each protein were determined with Image J software and compared as appropriate.

### Patients and tissue specimens

A cohort of 58 patients with primary OSCC receiving surgical treatment at our hospital from January 2007 to December 2012 were enrolled. This study protocol was reviewed and approved by the University Research Ethic Committee. Written informed consent was obtained from these patients. Patient inclusion criteria were listed as follows: (1) primary OSCC without any prior therapy; (2) patients underwent extensive tumor resection and neck dissection; (3) detailed information available including clinical, pathological and follow-up data. The archived H&E slides were retrieved and further analyzed to verify previous diagnoses. Eighteen normal oral mucosa were obtained from other non-cancer surgeries and histologically confirmed. Three paired fresh OSCC as well as adjacent healthy mucosa samples were obtained within 30 min after surgical resection and snap-frozen in liquid nitrogen and then stored under −80 °C until use.

### Histopathological evaluation and immunohistochemistry

The relevant clinicopathological parameters including histological grade, clinical stage, TNM classification, etc. were determined as described before [25, 26]. Immunohistochemical staining for LIN28B was performed on formalin-fixed, paraffin-embedded tumor specimens. Briefly, tissue sections from representative paraffin blocks were deparaffinised and rehydrated. Antigens retrieval was performed in microwave heating in citrate buffer (10 mmol/L, pH 6.0) for 20 min. These slides were further incubated with primary antibodies (LIN28B, Abcam, ab71415, 1:200 dilution) overnight and developed with 3,3′-diaminobenzidine and counterstained with hematoxylin. Negative controls (without primary antibody incubation) were included in each staining run. LIN28B immunoreactivity was evaluated independently by oral pathologists without knowledge of clinical data.

LIN28B immunoreactivity in both animal and human samples was semi-quantitatively evaluated according to staining intensity and distribution using the immunoreactive score which was calculated as intensity score × proportion score as we reported previously [25–27]. Intensity score was defined as 0, negative; 1, weak; 2, moderate; 3, strong. The proportion score was graded as 0, negative; 1, <10 %; 2, 11–50 %; 3, 51–80 %; 4, >80 % positive cells. LIN28B immunoreactivity in tissue samples was categorized into three subgroups based on the final scores: 0, negative; 1–4, low expression; 4–12, high expression.

### Statistical analysis

The quantitative data was shown as mean ± SD of two or three independent experiments and compared with Student’s *t* test or ANOVA. The associations between LIN28B abundance and clinicopathological parameters were evaluated using Fisher exact test. The overall survival rates were estimated using Kaplan–Meier method and compared with Log-rank test. The Cox proportional hazards model was applied to assess the impact of various clinicopathological parameters on patients overall survival. *P* values less than 0.05 (two-sides) were considered statistically significant.

## Results

### The expression pattern of LIN28B in DMBA-induced buccal OSCC animal model

**Fig. 1:**
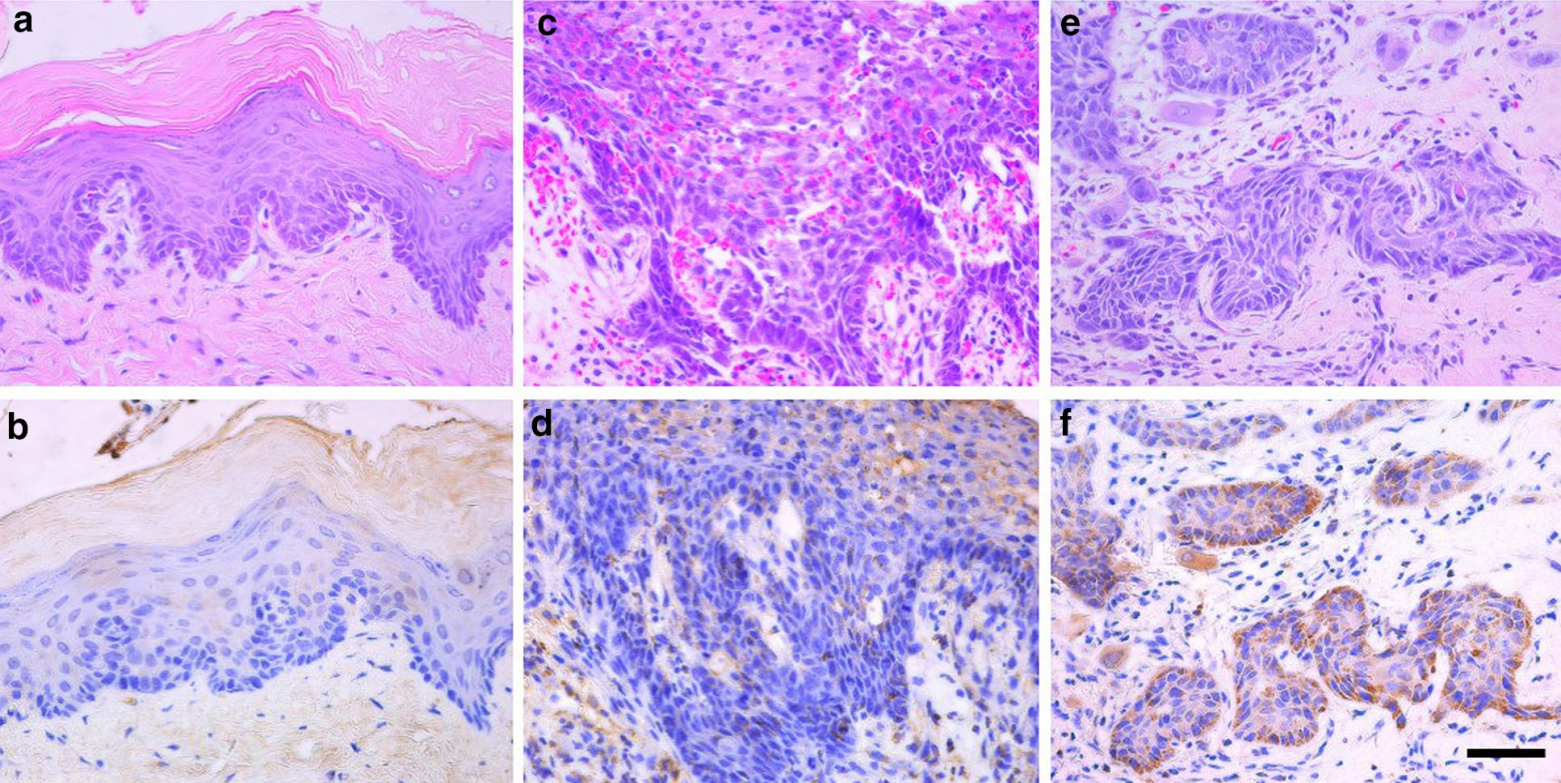
LIN28B expression in DMBA-induced OSCC animal model. **a**, **b** H&E staining and LIN28B immunohistochemical staining of normal buccal epithelial in control animals; **c**, **d** H&E staining and LIN28B immunohistochemical staining of epithelial dysplasia in experimental animals; **e**, **f** H&E staining and LIN28B immunohistochemical staining of squamous cell carcinoma in experimental animals. *Scale bar* 50 μm

We have provided strong evidence that the functional SNP (rs221636) in LIN28B gene contributes to oral cancer susceptibility in a Chinese population [21]. However, the oncogenic roles of LIN28B during oral cancer initiation and propagation in vivo have not yet been well established. To address this, we developed the hamster buccal pouch carcinogenesis model by chemical agent DMBA, which is a popular animal model for OSCC. Gross examinations of hamster buccal pouches following animal euthanization revealed no apparent changes in mineral oil-treated control animals, while thickened mucosa with rough surface was observed in 4- and 10-week DMBA-treated animals. Exophytic tumor-like or ulcer-like lesions were frequently visible in 16-week DMBA-treated animals. The histopathological analyses further confirmed the multiple pathological stages induced by DMBA such as epithelial dysplasia and invasive squamous cell carcinoma (Fig. 1a, c, e). Therefore, this animal model closely recapitulated the typical multiple stages of OSCC, reminiscence of human OSCC tumorigenesis [24]. We next determined the expression pattern of LIN28B in samples from this animal model. As shown in Fig. 1b, d, f, immunohistochemical staining of LIN28B in buccal samples from diverse stages of disease indicated negative in most normal epithelial but significantly higher in epithelial dysplasia and invasive carcinoma. Furthermore, our immunohistochemistry data (Table 1) revealed that LIN28B is overexpressed in the majority of SCC samples, while much less in samples with dysplasia and normal epithelial. Together, these data indicate that LIN28B might be critically involved in OSCC development and functions as a oncogenic driver behind oral tumorigenesis.

**Table 1:** LIN28B expression in the DMBA-induced buccal pouch squamous cell carcinogenesis model

	Lesion/total	LIN28B expression			*p* values
		Negative	Low	High	
Normal mucosa	10/10	7	2	1	0.0328
Dysplasia	9/10	5	3	1	
Squamous cell carcinoma	10/10	1	3	6	

### Expression and localization of LIN28B in OSCC cell lines

**Fig. 2:**
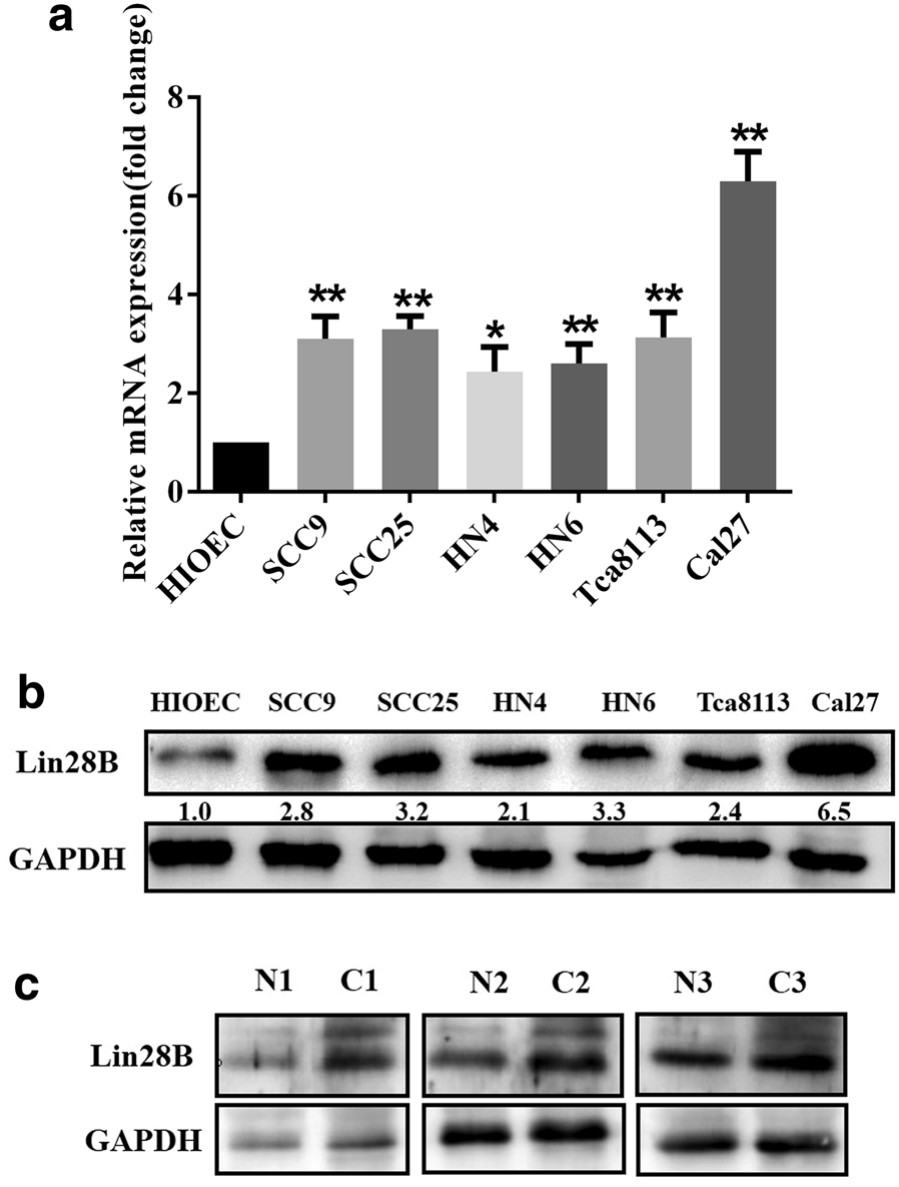
LIN28B expression in human OSCC cell lines and paired samples. **a** LIN28B mRNA levels in a panel of OSCC cell lines and HIOEC were measured by real time RT-PCR. **b** LIN28B protein levels in a panel of OSCC cell lines and HIOEC were measured by western blot. **c** LIN28B abundance in three pairs of OSCC and matched adjacent non-cancerous epithelial was compared by western blot. Data shown here are mean ± SD from three independent experiments, *P < 0.05, **P < 0.01, ANOVA and Student *t* analyses

**Fig. 3:**
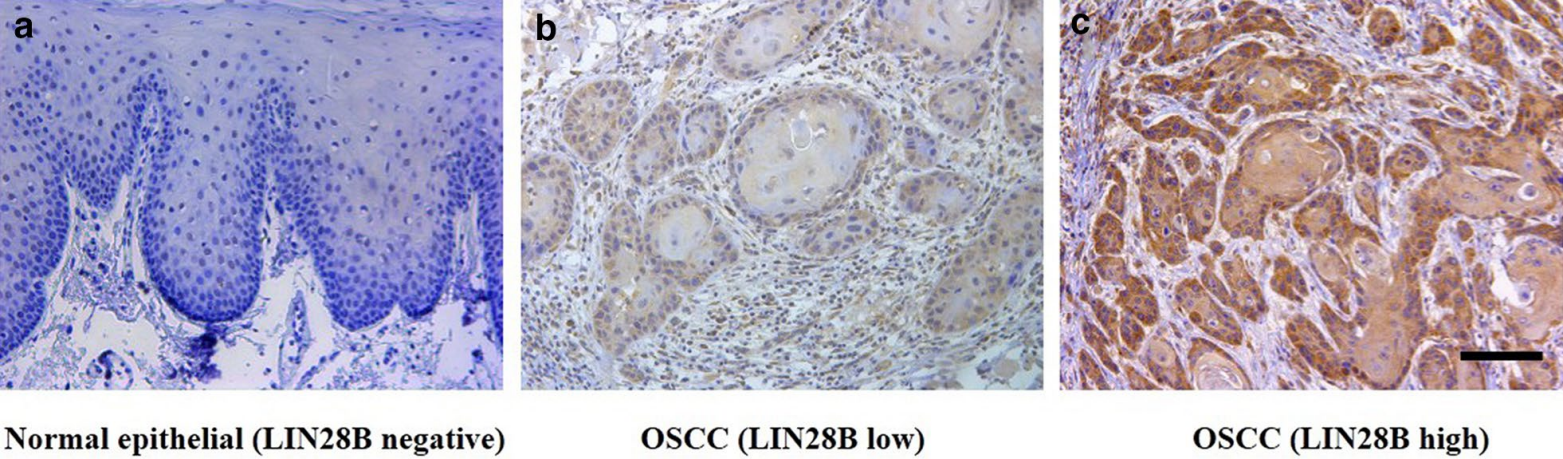
LIN28B expression in human OSCC samples measured by immunohistochemical staining. **a** Representative negative staining of LIN28B in normal oral epithelial; **b** Representative low expression of LIN28B in a primary human OSCC sample; **c** Representative high expression of LIN28B in a primary human OSCC sample. Nuclei are counterstained with hematoxylin. *Scale bar* 100 μm

To delineate the expression of LIN28B in human OSCC, both mRNA and protein abundance of LIN28B in a panel of OSCC cell lines were measured as compared to the immortalized oral epithelial cell line HIOEC. The real-time RT-PCR data revealed significantly increased LIN28B transcripts in all cancerous cells in relative to HIOCE (Fig. 2a). The western blot results further indicated remarkably upregulated LIN28B protein in all OSCC cell lines examined (Fig. 2b). In addition, Lin28B protein was pronouncedly increased in oral cancer tissues compared to the pair-matched adjacent non-cancerous tissues (n = 3) (Fig. 2c). We next performed cellular immunofluorescence assay in three selected cell lines to visualize the subcellular localization of LIN28B in OSCC. As displayed in Fig. 3, the LIN8B protein was mainly identified in nucleus, while much less in cytoplasm in OSCC cells.

### Clinicopathological characteristics and LIN28B expression in primary OSCC patients

To further examine LIN28B expression in clinical specimens and clinicopathological significance, we evaluated the expression level of LIN28B by immunohistochemical staining in a retrospective cohort of 58 primary OSCC patients. The epidemiologic information and clinicopathological parameters of these patients were listed in Table 2. In brief, 37 males and 21 females were enrolled with mean age 46.3 years. The follow-up durations ranged from 3 to 66 months (average 27.4 months).

**Table 2:** LIN28B expression pattern in OSCC and normal oral mucosa

	LIN28B expression			*p* values
	Negative	Low	High	
Normal oral mucosa	5	10	3	<0.0001
OSCC	0	19	39	

**Fig. 4:**
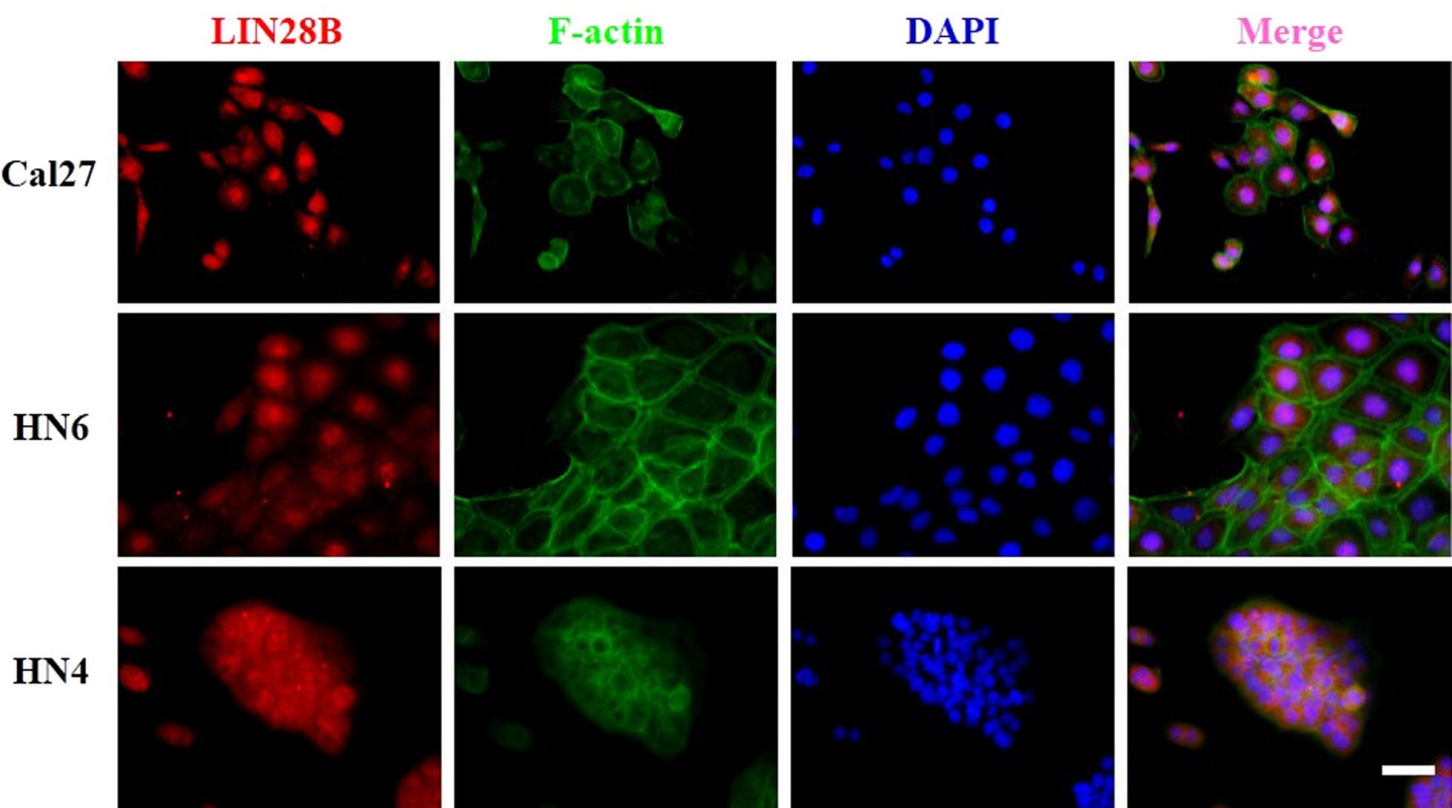
LIN28B subcellular distribution in OSCC cells determined by immunofluorescence. LIN28B is mainly identified in nucleus, less in cytoplasm in three OSCC cell lines. Nuclei and cytoplasm are counterstained with F-actin and DAPI, respectively. *Scale bar* 50 μm

As shown in Fig. 4c, LIN28B positive staining was identified in nucleus or both nucleus and cytoplasm in cancer specimens, whereas weak or negative staining was detected in the normal counterparts. Based on our immunohistochemistry scoring regime, LIN28B abundance in these primary OSCC and healthy oral epithelial was further categorized. As shown in Table 3, LIN28B levels in these OSCC sample can be graded as low (19) or high expression group (39), while negative (4), low expression (12) and high expression (4) in normal oral mucosa samples, thus indicating that LIN28B was aberrantly overexpressed in a fraction of oral cancers. The detailed relationships between LIN28B abundance and clinicopathological parameters were further shown in Table 2. There were no significant correlations found between LIN28B and patient age, gender, histopathological grade and clinical stage. Notably, LIN28B abundance was found to be associated with tumor size and cervical lymph nodes metastasis with *P* value 0.049, 0.0286, respectively.

**Table 3:** Associations between LIN28B expression and multiple clinicopathological parameters in primary OSCC

Clinicopathological parameters	Cases	LIN28B		*p* values
		Low	High	
Gender	58	19	39	
Male	37	12	25	1.000
Female	21	7	14	
Age				
≤60	30	13	17	0.0973
>60	28	6	22	
Tumor size				
T1–T2	31	14	17	0.0490
T3–T4	27	5	22	
Pathological grade				
I	33	12	21	0.5794
II–III	25	7	18	
Cervical node metastasis				
N(0)	30	12	18	0.2708
N(+)	28	7	21	
Clinical stage				
I–II	16	9	7	0.0286
III–IV	42	10	32	

### LIN28B expression levels associated with OSCC patients’ overall survival

**Fig. 5:**
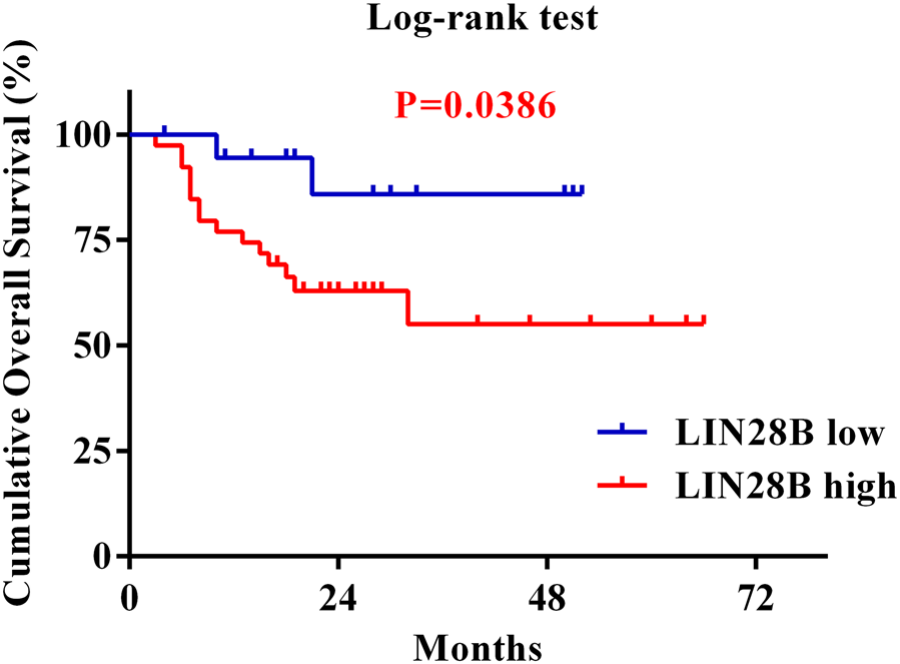
Kaplan–Meier graphs representing the probability of cumulative overall survival in OSCC patients based on LIN28B expression. High LIN28B expression significantly associated with reduced overall survival in OSCC patients. These survival analyses were estimated by Kaplan–Meier method and compared with Log-rank test

To further reveal prognostic values of LIN28B expression in OSCC patients, we next aimed to identify possible relationship between its expression and clinical outcomes. Until the last follow-up, 35 of 58 (60.3 %) patients remained alive and also disease-free, 6 (10.3 %) still alive but with local recurrences and/or cervical nodal metastases, whereas 17 (29.4 %) died due to post-surgical relapse, metastases or other diseases. The Kaplan–Meier survival analyses revealed that high LIN28B had adverse prognostic impact for patients’ outcomes. High LIN28B expression in OSCC significantly associated with short overall survival as compared to its low counterparts (Log-rank, *P* = 0.0386, Fig. 5).

To further assess prognostic values of LIN28B abundance for OSCC patients, both univariate and multivariate survival analyses were performed using Cox proportional hazards regression model. The univariate survival analysis identified no clinicopathological variables as key factors significantly affecting patients overall survival (Table 4). Multivariate survival analysis revealed that LIN28B expression status was a critical independent prognostic marker for overall survival of OSCC patients (*P* = 0.043, Table 4). Together, these data reveal that LIN28B can serve as an important prognostic biomarker for OSCC.

**Table 4:** Univariate and multivariate survival analyses for patients with OSCC

Variable	Univariate survival analysis			Multivariate survival analysis		
	Hazard ratio	95 % CI	*p* value	Hazard ratio	95 % CI	*p* value
Gender (male, female)	1.041	(0.385, 2.816)	0.937	1.228	(0.405, 3.722)	0.716
Age (≤60, >60)	1.276	(0.492, 3.308)	0.617	1.760	(0.597, 2.654)	0.306
Tumor size (T1–T2, T3–T4)	1.272	(0.490, 3.298)	0.621	0.450	(.083, 2.445)	0.355
Pathological grade (I, II–III)	0.536	(0.198, 1.454)	0.221	1.739	(0.616, 4.906)	0.284
Cervical nodal metastasis (N0, N+)	0.661	(0.251, 1.738)	0.401	0.370	(0.078, 1.770)	0.213
Clinical stage (I–II, III–IV)	1.770	(0.508, 6.162)	0.370	4.740	(0.638, 35.218)	0.128
LIN28B expression (low, high)	3.864	(0.883, 16.908)	0.073	4.905	(1.053, 22.846)	*0.043* *

* The numbers in italics indicate statistical significance

## Discussion

Accumulating evidence has revealed that the pluripotency factor LIN28B, a highly conserved RNA-binding protein, has emerged as a key player in many developmental and pathological processes including stem cell hemostasis, cellular metabolism and tumorigenesis [15, 28, 29]. Of great interests, it has been found to mediate cellular malignant transformation, fuel cancer cell proliferation, invasion and metastasis cascade as well as promote cancer stem cell maintenance in diverse human cancers [6, 10, 14]. Its overexpression significantly associated with cancer aggressiveness and poor prognosis [14, 15]. These findings indicate that LIN28B is an bona fide oncogene that might be exploited as cancer biomarker and therapeutic target. Here, we measured the expression patterns of LIN28B in OSCC cells, chemical-induced OSCC animal model and primary OSCC samples, and further determined its clinicopathological significance. Our findings reveal that LIN28B might be involved in OSCC initiation and progression by serving as an oncogene. Its overexpression associates with aggressive clinicopathological features and unfavorable patients’ prognosis in a significant fraction of OSCC.

The human oral tumorigenesis is characterized by multiple and consecutive histopathological stages from normal epithelial to invasive SCC driven by oncogenes activation and tumor suppressor inactivation [30]. This has been elegantly reproduced in chemical-induced animal models, such as DMBA-induced buccal SCC in hamster and 4-nitroquinoline 1-oxide (4NQO)-induced oral SCC in rodents [23, 24, 31]. In the present study, we utilized the former model and determined LIN28B expression during OSCC tumorigenesis by immunohistochemistry. As anticipated, our findings established that LIN28B expression was almost negative in normal epithelial, but its positive staining gradually became more and more in dysplasia and SCC samples, suggesting that LIN28B served as an oncogene critically involved in OSCC pathogenesis. This is well consistent with the oncogenic roles of LIN28B driving cancer initiation [11]. Because a line of evidence has revealed that LIN28B alone is capable to induce cell transformation in vitro and sufficient to initiate multiple cancers in vivo [11, 15–17]. Collectively, our data provide the in vivo evidence to support the oncogenic roles of LIN28B in oral tumorigenesis. Of course, further in-depth investigations into the detailed mechanism regarding LIN28B behind OSCC are warranted in future.

Previous findings have indicated that LIN28B is usually overexpressed in a broad spectrum of human cancers and is critically required for cancer overgrowth [15, 18]. Importantly, overexpression of LIN28B significantly associated with advanced stages, lymph node metastasis and unfavorable prognosis in diverse cancers including oral cancer [18, 20]. For example, Wu and his colleague reported that LIN28 and LIN28B expression levels were both increased in 72 primary OSCC and positively correlated with gender, tumor differentiation and patients’ survival [20]. Consistently, our data further extended these previous findings and determined LIN28B abundance in a panel of OSCC cell lines and an independent cohort of primary OSCC. Our results revealed that Lin28B was abnormally overexpressed in OSCC cells and human samples. Noticeably, elevated LIN28B significantly associated with tumor size and advanced clinical stages, but not the gender and tumor differentiation status as reported by Wu [20]. We reasoned that this discrepancy might be due to inherent tumor heterogeneity and different immunohistochemical scores and patient stratification. Interestingly, when the manuscript was in preparation, Lin and his colleague reported that elevated LIN28B correlated with lymph node metastasis in OSCC, although the mRNA abundance of LIN28B was utilized as readout by quantitative real-time PCR assay in 20 paired samples [32]. Therefore, our findings together with others strongly support the notion that LIN28B is aberrantly overexpressed in OSCC and significantly correlated with malignant clinicopathological features.

Although past decades have witnessed much progress in diagnosis and treatment for OSCC, however, the long-term survival still remains disappointing. Accurate prognostic prediction regime for OSCC is a great challenge, but highly beneficial in the clinic. Evidence has suggested that LIN28B may function as a key prognostic predictor for patients with colon, oesophagus cancer and neuroblastoma [17, 18, 33]. Indeed, our results indicated that high LIN28B correlated with decreased patients’ survival and served as an independent prognostic factor affecting patients’ survival. This finding is in agreement with previous report where they showed that patients with high LIN28B had earlier recurrence and decreased overall survival rates [20]. Thus, these data indicate that the expression status of LIN28B might serve as not only a novel diagnostic, but also a prognostic biomarker for OSCC. However, a large cohort of patients with long-term follow-up is still needed to definitively establish the prognostic values of LIN28B for OSCC.

In conclusion, our findings reveal that LIN28B might be critically involved in OSCC initiation and progression. Its upregulation correlates with aggressive clinicopathological parameters and adverse prognosis, thus suggesting that LIN28B might serve as a novel cancer biomarker as well as therapeutic target for OSCC.
